# Retraction Note to: Injectable hydrogel encapsulating Cu_2_MnS_2_ nanoplates for photothermal therapy against breast cancer

**DOI:** 10.1186/s12951-021-00780-0

**Published:** 2021-02-02

**Authors:** Ji‑jun Fu, Ming‑ yue Chen, Jie‑xia Li, Jun‑ hua Zhou, Sheng‑nan Xie, Ping Yuan, Bo Tang, Cheng‑ cheng Liu

**Affiliations:** 1 Department of Medical Oncology, The Fifth Affiliated Hospital of Guang-Zhou Medical University, Guangzhou Medical University, Guangzhou, 510700 China; 2 Key Laboratory of Molecular Target & Clinical Pharmacology, School of Pharmaceutical Sciences, Guangzhou Medical University, Guang-zhou, 511436 China; 3 Department of Biochemistry and Molecular Biology, Medical College of Shantou University, Shantou, 515041 Guangdong Province China; 4 School of Pharmaceutical Sciences, Jiangning District, China Pharmaceutical University, No. 639 Longmian Avenue, Nanjing, 211198 Jiangsu China; 5 School of Pharmacy, Nantong University, No. 19 Qixiu Road, Nantong, 226001 Jiangsu Province China

## Retraction Note to: J Nanobiotechnol (2018) 16:83https://doi.org/10.1186/s12951-018–0409-3

The authors have retracted this article [1] because of an error in the methodology. Recently, the authors have tested the Cu2MnS2 nanoparticles used in their study by XRD, XPS and TEM mapping and have found that the manganese element was not successfully doped. The composition of the nanoparticles was therefore CuS, not Cu2MnS2 as reported in the article. All authors agree to this retraction.
